# Tempo of magma degassing and the genesis of porphyry copper deposits

**DOI:** 10.1038/srep40566

**Published:** 2017-01-12

**Authors:** Cyril Chelle-Michou, Bertrand Rottier, Luca Caricchi, Guy Simpson

**Affiliations:** 1 Department of Earth Sciences, University of Geneva, Rue des Maraîchers 13, CH-1205 Geneva, Switzerland; 2 Univ. Lyon, UJM-Saint-Etienne, UBP, CNRS, IRD, Laboratoire Magmas et Volcans UMR 6524, F-42023 Saint Etienne, France

## Abstract

Porphyry deposits are copper-rich orebodies formed by precipitation of metal sulphides from hydrothermal fluids released from magmatic intrusions that cooled at depth within the Earth’s crust. Finding new porphyry deposits is essential because they are our largest source of copper and they also contain other strategic metals including gold and molybdenum. However, the discovery of giant porphyry deposits is hindered by a lack of understanding of the factors governing their size. Here, we use thermal modelling and statistical simulations to quantify the tempo and the chemistry of fluids released from cooling magmatic systems. We confirm that typical arc magmas produce fluids similar in composition to those that form porphyry deposits and conclude that the volume and duration of magmatic activity exert a first order control on the endowment (total mass of deposited copper) of economic porphyry copper deposits. Therefore, initial magma enrichment in copper and sulphur, although adding to the metallogenic potential, is not necessary to form a giant deposit. Our results link the respective durations of magmatic and hydrothermal activity from well-known large to supergiant deposits to their metal endowment. This novel approach can readily be implemented as an additional exploration tool that can help assess the economic potential of magmatic-hydrothermal systems.

Porphyry Copper Deposits (PCDs) are classically regarded as large upper crustal volumes of hydrothermally altered, veined and mineralized rocks centred on small appendices of magmatic rocks extruded from a larger magma body lying at greater depth^1^,^2^ (Fig. 1). A wealth of geologic and isotopic evidence demonstrates that metals (e.g. Cu, Mo, Au, Zn, Pb) and sulphur found at PCD crustal levels (i.e., 1–5 km depth) originate from degassing magma bodies (plutons) emplaced at 5–15 km depth^1^,^2^,^3^,^4^. A fundamental challenge in developing models for the genesis of PCDs is to understand the set of conditions leading to the formation of deposits spanning four orders of magnitude of copper endowment worldwide^5^. The genesis of the largest deposits is favoured by various factors, including long-lived magmatic-hydrothermal activity, high concentrations of “ore ingredients” (i.e., copper and sulphur) in the source magma, efficient partitioning and transport of these elements in the magmatic fluid, and localised precipitation of copper sulphides^6^,^7^,^8^. The common association of high-Sr/Y magmatic rocks with PCDs^9^ indicates that specific magmatic processes may generate particularly fertile magmas. Nevertheless, mass balance calculations for the Yerington and Bingham districts (two classical PCDs for which the size of the pluton is known) show that the volumes of the respective underlying plutons are sufficient to form the overlying PCDs, assuming “normal” initial copper and sulphur concentrations^10^,^11^. Furthermore, the compositions of high temperature single-phase intermediate-density fluids from barren and mineralized systems have broadly similar compositions^8^,^12^. These data suggest that the volume of magma delivering fluids from depth may exert a first order control on the metal endowment of PCDs^13^, and particularly metal-rich fluids are not essential for the genesis of economic ore deposits. Nevertheless, the study of the relationships between metal endowment and associated plutons is hindered by the lack of economic PCD in which the deeper portions of the systems are exposed (Fig. 1). In turn, if erosion has exposed the deeper roots of the magmatic system, the presence of an eroded PCD at shallower paleodepth is highly speculative (Fig. 1).

To explore the relative contribution of magma fertility, magma volume and rate of magma injection on the genesis and total metal endowment of PCDs, we simulated the emplacement, cooling and degassing of fluid-saturated magmatic intrusions of various volumes containing different initial concentrations of “ore ingredients”, emplaced in the crust over different timescales. In particular, we focussed on the tempo and the chemistry of the degassed fluid. Magma degassing is the process by which volatiles (e.g. water, sulphur, carbon dioxide), together with fluid-mobile elements (e.g. Cl, Cu, Pb, Zn) initially dissolved in magmas, exsolve and migrate to shallower depths where they may form a PCD or escape to the atmosphere (Fig. 1). The extraction of fluids and metals from magmas is the first critical step for the genesis of magmatic-hydrothermal ore deposits^1^,^2^ because it controls the amount of “ore ingredients” ultimately transferred to PCD formation levels (albeit their subsequent deposition is controlled by other factors). Therefore, quantifying the chemistry and rate of fluids released during the cooling of magmas in the Earth’s crust is key to identify magmatic systems with economic potential.

## Conceptual approach to model magma degassing

**Figure 2:**
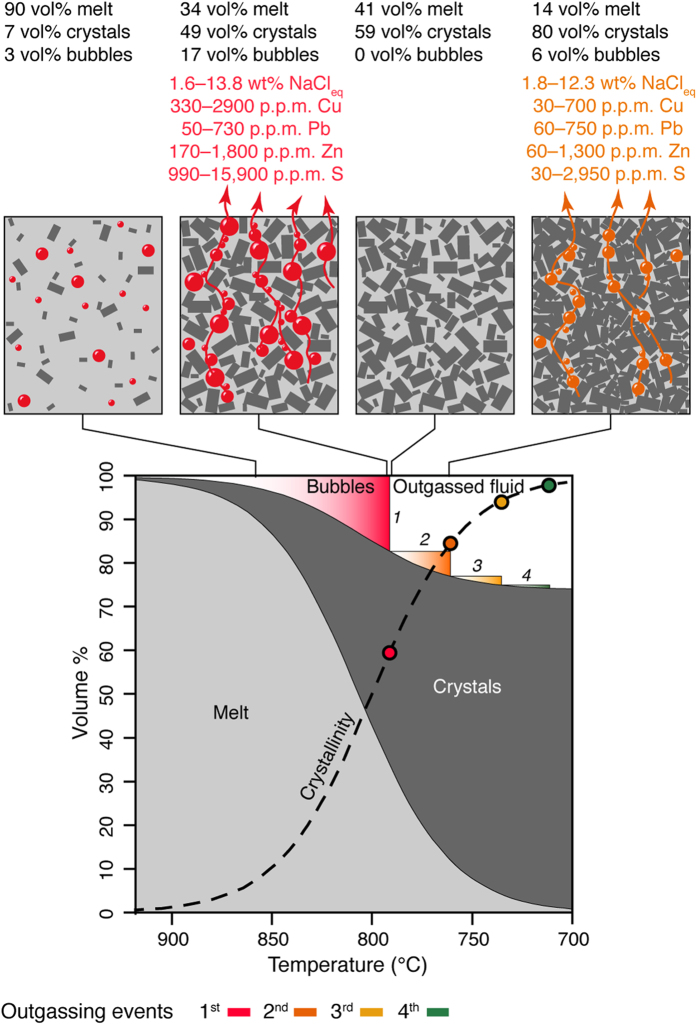
Melt, crystals and fluid volume variation as functions of temperature for a unit-volume of magma. The coloured fields show the relative gas volume released at each outgassing event. The fluid composition released during the first and second outgassing events calculated from Monte Carlo simulation are reported. Coloured circles on the crystallinity curve show the median magma temperature and crystallinity in correspondence of the four fluid outgassing events obtained from the simulations.

**Figure 3:**
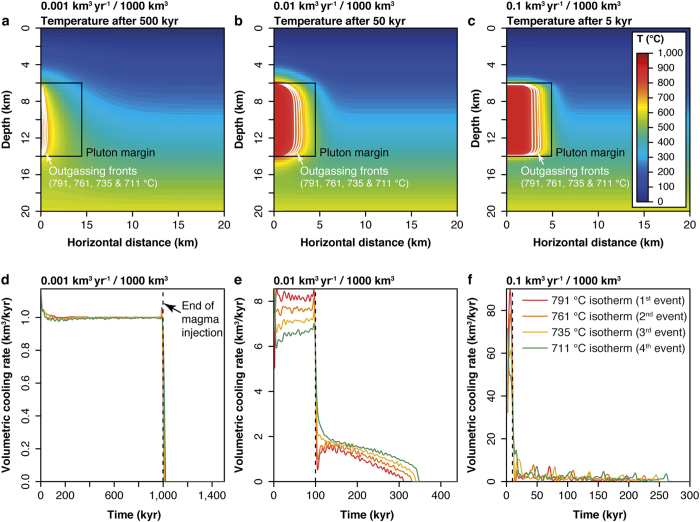
Thermal model outputs of incrementally emplaced plutons of 1000 km^3^ at three different fluxes. (**a–c**) Distribution of temperatures when half of the final volume of the pluton is emplaced with emphasis on the four outgassing fronts/isotherms. (**d–f**) Volumetric cooling rates of the incrementally emplaced plutons below the four isotherms.

Isobaric cooling of a thermally homogeneous volatile-saturated volume of magma leads to crystallisation and to the progressive exsolution of bubbles containing supercritical fluids (Fig. 2). Initially, the small size of the bubbles, the high viscosity of the crystal-bearing magma, and the formation of neutrally buoyant crystal-bubble aggregates may inhibit outgassing^14^,^15^,^16^,^17^. However, as cooling and crystallisation continue, the bubble volume fraction increases until a percolation threshold (*ϕ*_*c*_) is eventually reached at which bubbles coalesce and volatiles (and soluble metals) can escape the crystallising magma^18^. For a unit volume of magma this outgassing process occurs repeatedly in a series of pulses with decreasing amplitude as cooling and crystallization proceeds and continues until the magma is solid and the only fluids present in the system are those structurally bound to hydrous minerals (e.g., amphibole, biotite; Fig. 2). For this scenario, the outgassing events occur at fixed crystallinities (fixed bubble/residual melt ratio) and therefore at fixed temperatures (Fig. 2). As a consequence, for a thermally zoned magma intrusion cooling in the crust, the formation of permeability compatible with outgassing occurs simultaneously along isotherms corresponding to outgassing fronts (Fig. 3a–c).

## Modelling principles

In order to calculate the mass and chemistry of fluids outgassed from a magmatic system over time, we combined thermal modelling and Monte Carlo simulations, considering all uncertainty associated with the conceptual assumptions required to describe the exsolution of fluids from crystallising magma. We first simulated the chemistry and relative mass of fluids periodically outgassed from a fluid-saturated (initial H_2_O and CO_2_ contents of 5 wt% and 200 p.p.m., respectively) and thermally homogeneous unit-volume of magma that undergoes isobaric crystallization and cooling at 250 MPa (i.e., ca. 10 km). Because crystallinity, temperature, mass fraction of fluid, and chemistry of fluid at each outgassing event depend on various factors (i.e., *ϕ*_*c*_, fluid density, initial magma chemistry, and melt-crystal-fluid partition coefficients; see Methods) we contemporaneously and randomly varied all these parameters within reasonable ranges (Table 1) using a Monte Carlo approach to quantify the uncertainties of our results. A total of 100,000 simulations were performed assuming uniform probability distributions for each of the considered parameters (Supplementary Fig. S1). The first four outgassing events (i.e., at ca. 791, 761, 735 and 711 °C; Fig. 2) account for a cumulative loss of ca. 98% of the total volatiles present in a unit-volume of magma (Fig. 2, Supplementary Fig. S1a), and are therefore the only outgassing events considered hereafter.

To quantify the mass and composition of fluids released from cooling magma intrusions over time, the thermal models were analysed to retrieve the volume of magma that cooled below these isotherms as function of time. We modelled the thermal evolution of fluid-saturated dioritic-granodioritic magmatic bodies emplaced at an average depth of 10 km (i.e., ca. 250 MPa) with an initial temperature of 900 °C. Unlike previous models that considered ‘instantaneous’ emplacement of large magma volumes^10^,^11^,^19^,^20^, we draw on modern views of plutons building^21^ by modelling the incremental assembly of magmatic systems in the upper crust at magma injection rates between 0.001 and 0.1 km^3^ yr^−1^ and final volumes ranging from 100 to 10,000 km^3^. Different emplacement geometries and initial injection temperatures were tested and the results are essentially identical to those on which our main conclusions are based (see Methods). For each thermal model, we extracted the respective volumes of magma that cool below one of the four percolation isotherms in between each time step (Fig. 3d–f; Supplementary Fig. S2). Subsequently, we used these volumetric cooling rates (based on incrementally built plutons) along with the Monte Carlo simulations (based on a unit-volume of magma) to estimate the total mass flux and composition of the outgassed fluid through time (see Methods). The flux and the chemical composition of the bulk fluid outgassed from the emplaced magma body at each time step was calculated as the sum and the weighted mean of each on the fluids outgassed along the four outgassing fronts, respectively.

## Results and Discussion

### Fluid flux and fluid composition

**Figure 4:**
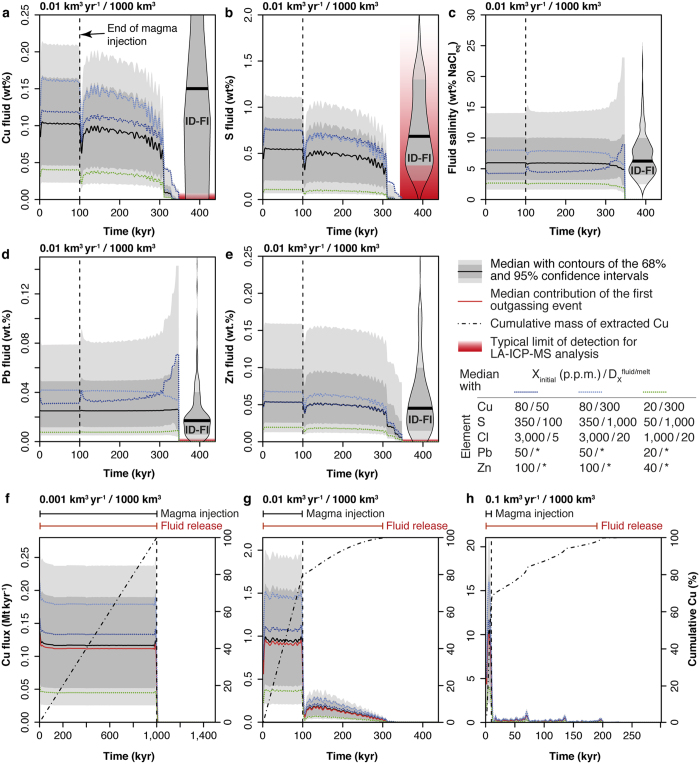
Temporal evolution of the fluid chemistry and flux of copper released from a representative magmatic intrusion of 1000 km^3^ volume at specified flux. (**a–e**) Composition of the fluid exsolved from a magma intrusion emplaced at a rate of 0.01 km^3^ yr^−1^ compared to the composition of natural intermediate-density fluid inclusions (ID-FIs) measured at PCDs worldwide (from ref. 23) plotted as beans. (**f**–**h**) Flux of copper outgassed from magmatic intrusions emplaced at different rates. The black and red capped lines on top of the panels provide the duration of magma injection and fluid release, respectively. * In the legend indicates that the fluid/melt partition coefficients for zinc and lead are linked to the chlorinity of the fluid (see Methods).

Results show that the composition of the fluids released over time from the magmatic intrusion is largely controlled by the chemistry of the fluids outgassed at the highest temperature when magma reaches the first percolation threshold (at ca. 791 °C; Fig. 4, Supplementary Figs S3–10). This is because the first outgassing event accounts for 50–75 wt% of the total fluid ultimately released from a given volume of magma (Supplementary Fig. S1a). As a result, as long as there is a component of the fluid outgassed at the first percolation threshold (ca. 791 °C) the composition of the bulk outgassed fluid remains essentially stable. Only during the latest stages of magma crystallization, the metal, sulphur and chlorine content of the fluid may increase (D^fluid/melt^ < ~20) or decrease (D^fluid/melt^ > ~20) depending on their respective fluid/melt partition coefficients^22^. However, these latest stages of outgassing are of very limited importance from an ore-forming perspective because they represent negligible amounts of fluids, which are furthermore depleted in sulphur and copper (Supplementary Fig. S1). Therefore, the duration of the ore-forming hydrothermal activity was assumed to correspond to the duration of fluid release that incorporates a component outgassed at the first percolation threshold (i.e., at ca. 791 °C; Supplementary Table S1).

For all simulations, the chemistry of the computed fluid matches the salinity (2–15 wt% NaCl_eq_), lead content (0.005–0.08 wt%), zinc content (0.01–0.16 wt%) and sulphur content (0.1–1.1 wt%) of intermediate-density fluid inclusions (ID-FIs) measured in PCDs^23^ (Fig. 4b–e; Supplementary Figs S3), which are the best proxy for the pristine fluids released from crystallising magmas (i.e., before chemical modification through fluid rock interaction, precipitation and/or boiling). We note that the high limit of detection for sulphur in ID-FIs (typically 0.2–2.5 wt%) coupled with big uncertainties (typically 20–50% relative) likely biases the record toward slightly higher average values with respect to what we calculate (Fig. 4b). Alternatively, some of these high measured values may correspond to sulphur input from mafic magmas, something not accounted for in this study. In the case of copper, our model predicts concentrations of 0.02–0.21 wt% similar to the most abundant measurements of ID-FIs from PDCs. However, the calculated range of Cu concentration is much narrower and toward the lower end of the measured values (Fig. 4a; Supplementary Fig. S4). This is possibly due to the well-documented post-entrapment diffusion of Cu into quartz-hosted fluid-inclusions^24^. Interestingly, for the range of initial compositions investigated (Table 1), the amount of sulphur transported by the fluid is on average five times higher than copper (Fig. 4a,b; Supplementary Figs S4, 5, 8 and 9). In more than 80% of the simulations S/Cu_fluid_ > 2 and enough sulphur is present in the fluids to precipitate all the copper together with large amounts of pyrite and other sulphides, as typically observed in PCDs. Therefore, our results suggest that mafic magma input, proposed to critically provide the sulphur required to form PCDs^25^,^26^,^27^, although being helpful, may not to be necessary. Our results also show that, due to their high fluid/melt partition coefficients (Table 1), the main control on the fluid metals and sulphur concentrations is their initial concentrations in the magma (Fig. 4). Therefore, while normal magmas (i.e., with no special metal and sulphur enrichment) can form an economic PCD, high initial sulphur and metals concentrations in the magma significantly increase the probability of forming a deposit.

### Time scales of degassing and porphyry copper formation

**Figure 5:**
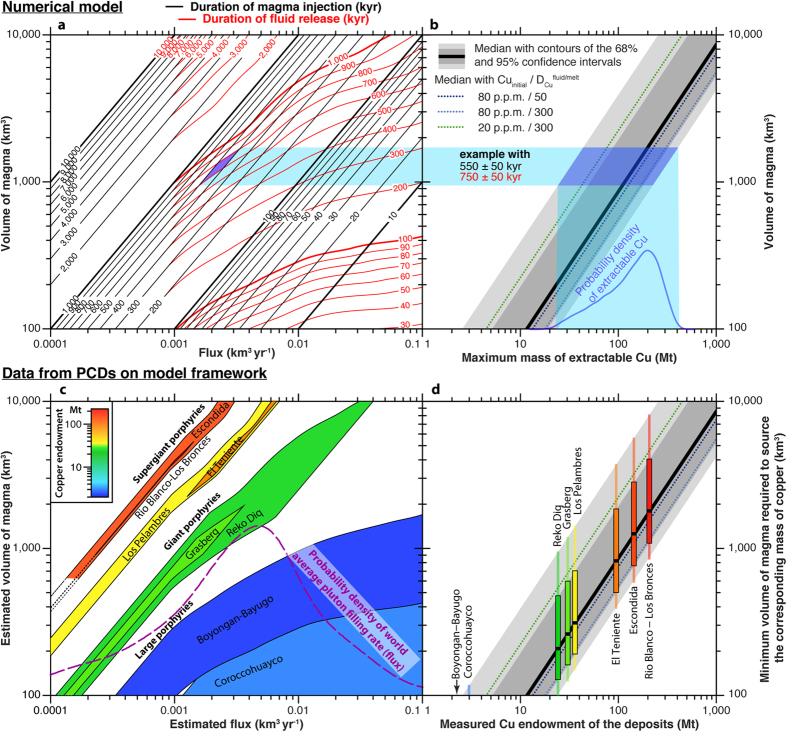
Summary of the model results and comparison with natural PCDs. (**a**) Contours of durations of magmatic activity (black) and ore-forming fluid release (red). (**b**) Maximum mass of extractable copper obtained from Monte Carlo simulations for varying magma volumes. The blue field in **a** represents the intersection area of durations of the ore-related magmatic activity (550 ± 50 kyr) and of the ore-forming fluid released (750 ± 50 kyr) from a hypothetical system. The estimated magma volume for this system is subsequently used in (**b**) to provide a probabilistic estimate (blue curve) of the maximum mass of copper that could be extracted during degassing. (**c**) Results of the inversion on well-dated porphyry copper deposits of known copper endowments based on (**a**). The dashed curve corresponds to the density distribution of long-term averaged pluton filling rates measured worldwide (data from ref. 28). (**d**) Independent estimates of the minimum magma volume required to provide the known amount copper of the deposits. Probability density functions on **b** and **c** are dimensionless along the Y axis (relative probability density) and has no relation to the Y axis of **b** and **c**, respectively.

The rate of magma emplacement in the crust controls the duration of fluid, metals and sulphur release, with respect to the duration of magmatism (Fig. 4f–h, Supplementary Figs S8–10). At low magma fluxes (≤0.001 km^3^ yr^−1^) all the injected magma is cooled to solidus and all the fluids are released during the magma injection phase. At higher magma fluxes, although most outgassing occurs during injection, a significant mass (up to 30%) of the total magma remains at temperature higher than 800 °C (i.e. temperature at the onset of outgassing; Fig. 2), and keeps degassing long after injection has ceased (Fig. 4f–h). Considering a fixed rate of heat release into the host rock and equal final volume of injected magma, with increasing rates of magma input the total amount of heat released at the end of the episode of magma injection decreases. Thus, reservoirs accreted with larger magma fluxes cool for a longer period from the end of magma input. Additionally, at relatively high fluxes (>0.001 km^3^ yr^−1^), the volumetric cooling rates sharply decrease just after injection ceases. This is due to a combination of latent heat release (which buffers the thermal state of the intrusion) and to the cessation of displacement of the host rocks (advection) caused by the intrusion. At any given final magma volume, the ratio between the duration of fluid release and the duration of magma emplacement increases with increasing magma flux (Figs 4f–h and 5a; Supplementary Table S1). Assuming that both of these timescales can be measured, we propose that they can be used to estimate the average magma flux, the final magma volume and the probability distribution of the maximum copper endowment of PDCs (Fig. 5a,b). Note however, that this simple approach neglects other potentially important factors such as the efficiency of copper extraction during degassing, focussing of fluids, precipitation of sulphides, or preservation of the deposit. Consequently, our approach can only provide an upper limit of potential copper endowment.

To test our method, we compiled radioisotopic geochronological data from large (2–3 Mt Cu; Corroccohuayo, Peru and Bonyogan-Bayugo, Philippines), giant (20–40 Mt Cu; Grasberg, Papua, Reko Diq, Pakistan and Los Pelambres, Chile) and supergiant (100–200 Mt Cu; El Teniente, Escondida and Rio Blanco-Los Bronces, all in Chile) PCDs for which the actual copper endowment is known (Supplementary Fig. S11). The duration of ore-related magmatism was estimated by considering the difference between the U-Pb zircon emplacement age of the oldest and the youngest ore-related magmatic rocks, except for the Grasberg deposit where only biotite ^40^Ar/^39^Ar dates were available. The duration of the ore-forming fluid release was calculated by the difference between the U-Pb zircon emplacement age of the oldest ore-related magmatic rock and the youngest molybdenite Re-Os age. Uncertainties inherent to isotopic ages were propagated by quadratic addition onto the uncertainties of the durations. It is noteworthy that these durations (of both ore-forming magmatic and hydrothermal activity) only represent minimum values while likely providing close estimates (see Methods). Based on these durations, we estimate the magma volume and average magma flux associated with the formation of these deposits (Fig. 5c). Overlapping durations of magmatic and hydrothermal activities, that mostly reflect high analytical uncertainties, result in correspondingly large uncertainties of the estimated volume and flux. Within the range of initial magma composition and values of partition coefficients that we considered (Table 1), we also independently estimated the minimum volume of magma required to provide these deposits with their known mass of copper (Fig. 5d).

At an average magma flux similar to the long-term-averaged filling rates measured for plutons worldwide (i.e., 0.001–0.01 km^3^ yr^−1^; ref. 28), the duration-based volume estimates correlate with the known copper endowment of the deposits, with small (~100 km^3^) and large (~5,000 km^3^) plutons corresponding to large and supergiant deposits, respectively (Fig. 5c). This estimated volume (Fig. 5c) tends to be systematically higher than, but overlapping with, the minimum magma volume required to explain the amount of copper in the deposits (Fig. 5d). This shows, as previously suggested^11^, that the processes of copper extraction and precipitation in PCDs are not 100% efficient.

We show that the duration of magmatic and hydrothermal activity can be used to quantify the magnitude of maximum potential copper endowment of PCDs. Nevertheless, the actual efficiency of ore precipitation and ore preservation cannot be quantified using this approach. Our method requires good knowledge of the ore-related magmatic history of the deposit, high-precision zircon U-Pb emplacement ages of all ore-related magmatic phases, together with a representative set of molybdenite Re-Os ages to bracket the duration of the ore-forming fluid release. Interestingly, our results confirm that large deposits (containing copper on the order of few million tons or less) form on timescales smaller than 100 kyr (Fig. 5; refs 29 and 30), often at the limit of current state-of-the-art analytical precision in geochronology. This makes them ‘geochronologically’ undistinguishable from the small deposits (<0.1 Mt of copper). The first order approach we propose provides explorers with a new tool that may help identifying the largest deposits (>20 Mt of copper), better target exploration and assist decision-making. Improvement of analytical precision and accuracy of the U-Pb and Re-Os radioisotopic systems are instrumental to the determination of durations at fine timescales and will improve the precision of the estimated magma volume, magma flux and mass of extractable copper.

### Implications for arc magmatism

Our results confirm that almost any hydrous intermediate arc magma has the potential to exsolve fluids capable of forming a PCD. The agreement between our modelling results and the data collected from PCDs suggests that, regardless of the range of initial metal and sulphur concentrations in the magma, long durations of a magmatic episode (>1 Ma) and large intrusion volumes (>1,000 km^3^) are key requirements for the genesis of the largest economic deposits (>20 Mt of copper; although the specific mechanisms for generating such large volumes of magma remain unclear). These findings are in line with global estimates of PCD formation rates (231 deposit/Myr; ref. 31), which suggest that >20% of the ca. 1075 active or potentially active arc-related volcanic centres (Global Volcanism Program: http://volcano.si.edu) are forming a PCD at depth today (assuming magmatic system lifespans >1 Myr). Similarly, a comparable proportion of outcropping plutons may have once had a now eroded PCD on top. This reinforces the idea that PCD provinces mainly correspond to optimal erosion levels rather than more PCD-productive crustal segments^32^,^33^.

## Methods

### Thermal model

We obtained the temperature evolution within a given magmatic body and the surrounding rocks by solving:


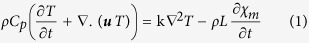


where *T* is the temperature (K), 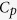 is the heat capacity (J kg^−1^ K^−1^), 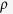
is the density (kg m^−3^), 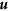 is the solid velocity vector caused by intrusion (m s^−1^), k is the heat conductivity (W m^−1^ K^−1^), L is the latent heat of fusion (J kg^−1^) and 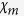
is the melt fraction. This equation considers transient heat exchange due to advection and conduction along with latent heating due to crystallisation within the cooling magma. Advective heat transfer by fluid circulation in the permeable crust around the pluton has been assumed to have a minor effect on pluton cooling at least to its solidus (e.g., ref. 34) and was therefore not considered for simplicity. It is likely that in the case of a highly permeable crust, our model would underestimate the cooling rate and overestimate the duration of outgassing (e.g., ref. 35). The melt fraction is given by:


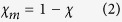


where 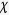 is the crystal volume fraction in the system crystals + melt, and was considered to vary with temperature (*T*, °C) as:


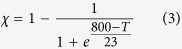


Equation (3) provides the best fit to the data of ref. 36 collected for granodioritic compositions at a confining pressure of 200 MPa and water-saturated conditions. Solutions were obtained assuming an axisymmetrical geometry using the Galerkin finite element method on 4-node quadrilaterals with linear shape and weighting functions. All calculations were performed with the following physical parameters: initial geothermal gradient 30 °C^ ^km^−1^, intrusion centre depth 10 km, heat conductivity 2.7 W^ ^m^−1^ K^−1^, heat capacity 1 kJ^ ^kg^−1^ K^−1^, rock density 2700 kg^ ^m^−3^, latent heat of fusion 350 kJ^ ^kg^−1^.

Most calculations were performed assuming a horizontally expanding cylindrical magmatic system (thickness of 8 km) with new magma injection (vertical cylinder) occurring in the centre of the intrusion with a temperature of 900 °C. We also tested sensitivity to the intrusion geometry and injection temperature by comparing these results with outward inflating spheres (new magma injection in the form of spheres) with an injection temperature of 1,000 °C (Supplementary Table S1). We studied a range of volumetric intrusive fluxes (0.001–0.1 km^3^ yr^−1^) and final injection volumes (100–10,000 km^3^). Intrusion was treated by setting the temperature within the newly intruded portion of the magma body (900 °C or 1,000 °C), while the adjacent rocks were advected outward to accommodate the volume increase. However, because the model is formulated in the Eulerian framework, advection does not influence the computational mesh, which remains fixed. The velocity field (with components 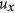 and 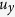) for the horizontally expanding cylinders was computed as 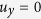, 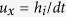 for 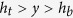, otherwise 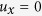, where 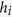 is the thickness of newly intruded cylinder each time step, 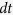 is the duration of the time step and 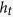 and 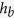 are the y coordinates of the top and bottom of the intrusion, respectively. Zero horizontal heat flux was imposed on lateral boundaries. These boundaries were set at a distance sufficient to not influence the temperature of the cooling intrusion. A constant heat flux in the vertical direction was imposed on the base. This heat flux was chosen to be in equilibrium with a linear initial geothermal gradient (of 30 °C^ ^km^−1^).

On the basis of the computed temperature field (*T(x, y, t*)), we back calculated the volume of the pluton cooling through four specific isotherms (representing four different outgassing fronts) through time (Supplementary Fig. S2). These magma volumes were then used to reconstruct the fluid release history as described in the following section.

### Monte Carlo simulations

We considered four sequential events of fluids release (n = 1–4). Outgassing occurs once the volume fraction of fluid with respect to the melt phases reaches the percolation threshold (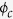, volume fraction):


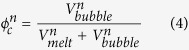


where 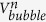 and 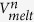 are the volumes (km^3^) of bubble and melt at the percolation threshold 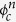, respectively, and can be expressed as:


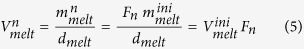



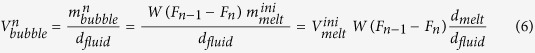


where: 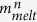 and 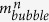 are the masses (in Mt) of bubble and melt at each percolation threshold, 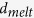 and 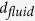 (Mt km^−3^) are the densities of melt and fluid, respectively, 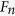 is the mass fraction of melt remaining at outgassing event n, 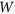 is the mass fraction of water dissolved in the melt, and 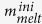 (Mt) and 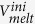 (km^3^) are the initial mass and volume of injected magma, respectively. Note that 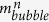 corresponds to the mass of fluid outgassed at the event n. Combining equations (4), (5) and (6) we obtain the mass fraction of melt at each outgassing event:


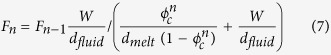


with 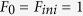. The crystallinity (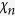, in volume fraction) at outgassing event n is:


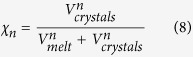


where 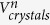 (km^3^) is the volume of crystals at the outgassing event n:


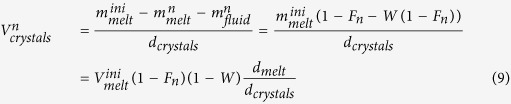


where 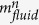 is the cumulative mass of exsolved fluids (the sum of the mass of already outgassed fluid and the mass of fluid bubbles trapped in the magma). Combining equations (5), (8) and (9) yields:


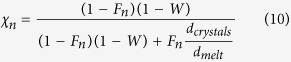


The chlorine, copper, lead, zinc and sulphur concentrations of the melt, crystals and fluid were calculated assuming equilibrium partitioning between these phases. The concentration of element x in the melt at the outgassing event n (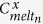, wt%) is:


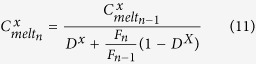


with 
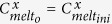
 and where 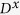 is the bulk partition coefficient of element X between crystals + fluid and the melt:


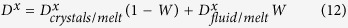


where 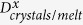 and 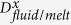 are the partition coefficients of element x between crystals and melt and fluid and melt, respectively. The concentration of element x in the fluid at outgassing event n (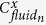, wt%) is:


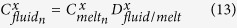


Fluid salinity (wt% NaCl_eq_) was derived from the chlorine content as follows:


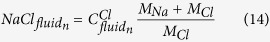


where 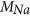 and 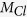 are the molar masses of sodium and chlorine, respectively.

### Fluid flux and fluid composition through time

For an incrementally growing magmatic system, the total mass of fluid outgassed at each time interval (m_*fluid*_ (*t*) in Mt) is calculated as the sum of the fluids outgassed along the four outgassing fronts. Using equation (6) it yields:


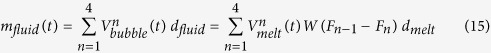


where 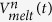 (in km^3^) is the volume of melt that cooled below one of the four percolation isotherm between two time steps, extracted from the thermal model. Similarly, the total mass of element x being outgassed from the system at each time step is:


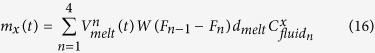


Finally, at each time step, the composition of the bulk fluid for element x is given by:


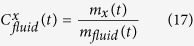


### Choice of input parameters

The range of initial melt concentrations of chlorine, copper, lead, zinc and sulphur (Table 1) was derived from a compilation of melt inclusion data from andesitic to rhyolitic arc-related volcanic rocks (Supplementary Table S2). We note that sulphur concentrations between 20 and 500 p.p.m. are compatible with solubility data for water-saturated andesitic to rhyolitic melts at typical oxygen fugacities of NNO to NNO + 2 (where NNO is the nickel-nickel oxide buffer)^37^.

The upper limit of 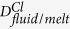 was calculated from the pressure dependent calibration of ref. 38 for haplogranitic magma at 250 MPa. The lower value was set to 3, consistent with experimental data on andesitic melts at 200 MPa (refs 39, 40, 41). Experimental 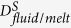 values for andesitic to rhyolitic composition are mostly between 30 and 1700, and include values obtained at sulphide or sulphate saturation^42^. Data published for 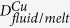 vary from ca. 20 to more than 500 for typical S- and Cl-bearing arc-related systems^43^,^44^. These studies highlight the variety of possible Cu-complexing ligands (S and Cl species) in the magmatic-hydrothermal environment, which does not allow to simply link copper partitioning with the salinity of the fluid. However, there is no model yet available to calculate 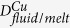 as a function of Cl and S fluid concentrations. On the contrary, 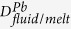 and 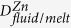 were found to correlate positively with the fluid chlorinity in Cl- and S-bearing systems^43^. Accordingly, the fluid/melt partitioning of lead and zinc are slaved to the chlorinity of the fluid (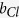 in mol/kg of solution) at each percolation threshold:


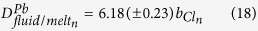



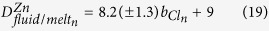



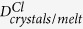
, 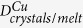, 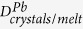 and 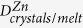 have been compiled from the GERM database (http://earthref.org/GERM/) for plagioclase, amphibole, biotite, K-feldspar and magnetite. We have assumed that sulphur is fully incompatible in these minerals. We have calculated the bulk crystals/melt partition coefficients for Cl, Cu, Pb and Zn for various mixtures of plagioclase, amphibole, biotite, K-feldspar, quartz and magnetite. This results in values from 0 to 0.55 for 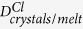, from 0.2 to 1.5 for 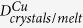, from 0.2 to 1.0 for 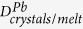 and from 0.4 to 3.7 for 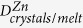.

Density of aqueous fluids is a function of temperature, pressure and composition. The upper limit of fluid density was computed at 750 °C, 250 MPa and 10 wt% NaCl, and the lower limit at 800 °C, 250 MPa and 0 wt% NaCl, using the Sowat software^45^. The resulting densities range from 0.497 g^ ^cm^−3^ to 0.612 g^ ^cm^−3^, consistent with densities estimated in natural intermediate-density fluid inclusions (ID-FIs; ref. 23).

Finally, the range of values for the percolation threshold were extracted from refs 15 and 18.

### Geochronological data from PCDs

Data are from ref. 46 for Rio Blanco–Los Bronces, ref. 47 for La Escondida, refs 48, 49, 50 for El Teniente, ref. 51 for Los Pelambres, ref. 52 for Grasberg, ref. 53 for Reko Diq, ref. 30 for Coroccohuayco, and refs 54 and 55 for Boyongan–Bayugo. The magmatic-hydrothermal footprints of the underlying plutonic roots may be in the form of spatially distinct magma plugs and ore bodies over an area commensurable to the extent of the pluton at depth^11^. Therefore, in order to calculate the duration of the full magmatic-hydrothermal system at each porphyry copper system, geochronological data from nearby (sometimes overlapping) coeval deposits were treated together as long as they are in temporal continuity (i.e., not separated by a statistically significant temporal gap). Data for the Rio Blanco–Los Bronces district include those from the Rio Blanco, Don Luis, Sur Sur, La Americana, San Enrique-Monolito, and Los Sulfatos deposits. At the Escondida district, data from the Escondida, Escondida Este, Pampa Escondida, Escondida Norte and Zaldivar were treated as a whole. At the Los Pelambres district, data from the Los Pelambres and Frontera deposits were treated together. Data from the Grasberg and Ertsberg deposit were treated together for the Grasberg district. Finally, at the Reko Diq district, data from the H14 and H15 centres were treated as a whole. We note that the estimated durations are associated with some assumptions. In fact, the reported crosscutting relationships at these PCD deposits do not always allow to confirm the relative timing between the youngest Re-Os molybdenite age and the age of youngest ore-related porphyry intrusion. We have assumed that (1) the latest ore-related magmatic rock at PCD exposure level closely correspond to the last pulse of magma injection in the incrementally building upper crustal pluton, (2) that the ore-forming fluids started outgassing immediately after the emplacement of the first ore-related porphyry (dyke or stock), and (3) that the youngest molybdenite Re-Os date represents the best approximation for the age of the youngest mineralization. For the sake of testing our model and providing orders of magnitude results in term of magma volume and intrusive flux, we argue that these are reasonable assumptions.

## Additional Information

**How to cite this article**: Chelle-Michou, C. *et al*. Tempo of magma degassing and the genesis of porphyry copper deposits. *Sci. Rep.* **7**, 40566; doi: 10.1038/srep40566 (2017).

**Publisher's note:** Springer Nature remains neutral with regard to jurisdictional claims in published maps and institutional affiliations.

## Supplementary Material

Supplementary Figures

Supplementary Table 1

Supplementary Table 2

## Figures and Tables

**Table 1:** Parameter ranges used in Monte Carlo simulations.

		min	max
Partition coefficients	D^fluid/melt^ Cl	3	32
	D^fluid/melt^ Cu	20	500
	D^fluid/melt^ Pb	*Linked to chlorinity*	
	D^fluid/melt^ Zn	*Linked to chlorinity*	
	D^fluid/melt^ S	30	1700
	D^crystal/melt^ Cl	0	0.55
	D^crystal/melt^ Cu	0.2	1.5
	D^crystal/melt^ Pb	0.2	1.0
	D^crystal/melt^ Zn	0.4	3.7
Initial concentrations in the melt (p.p.m.)	Cl	800	4500
	Cu	10	100
	Pb	10	70
	Zn	20	150
	S	20	500
Density (Mt/km^3^)	fluid	497	612
	crystals	2700	
	melt	2400	
Percolation threshold (*ϕ*_c_, vol%)		20	35

See the Methods section and Supplementary Table S2 for the justification of these parameters.

**Figure 1:**
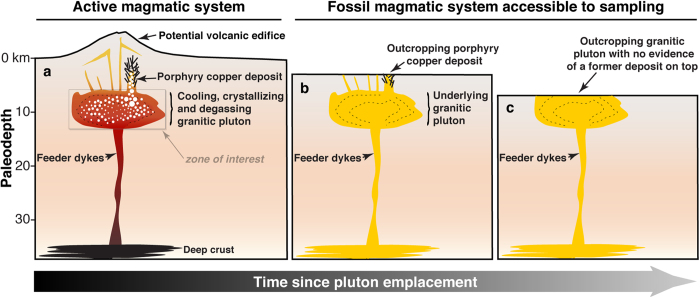
Architecture and temporal evolution (a to c) of a typical arc-related magmatic-hydrothermal system. The grey box in panel (**a)** shows that this study puts emphasis on the intrusive magmatic bodies responsible for the supply of mineralizing fluids.
