# Supplementary material for: Tempo of magma degassing and the genesis of porphyry copper deposits
Source: Sci Rep. 2017 Jan 12;7:40566. doi: 10.1038/srep40566 (PMC5227963; doi:10.1038/srep40566)
Supplement: Supplementary Figures [file srep40566-s1.pdf]

SUPPLEMENTARY FIGURES

Tempo of magma degassing and the genesis of porphyry copper deposits

Cyril Chelle-Michou[1,2]*, Bertrand Rottier[1], Luca Caricchi[1] and Guy Simpson[1]

[1] Department of Earth Sciences, University of Geneva, Rue des Maraîchers 13, CH-1205 Geneva, Switzerland.

[2] Univ. Lyon, UJM-Saint-Etienne, UBP, CNRS, IRD, Laboratoire Magmas et Volcans UMR 6524, F-42023 Saint Etienne, France

*Corresponding author: C.C.-M. cyrche@gmail.com

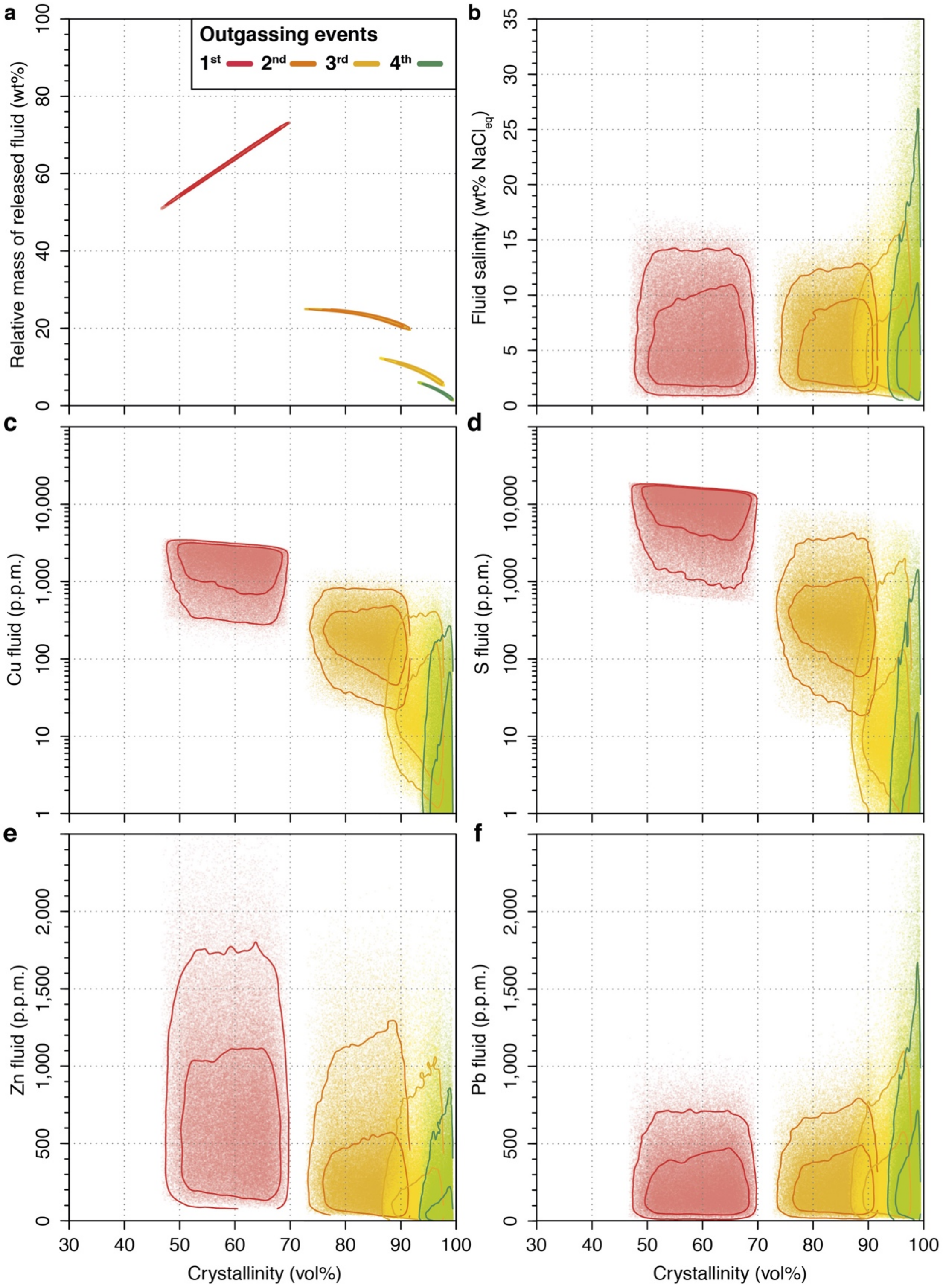

Supplementary Figure S1 | Results of Monte Carlo simulations for a crystallising unit volume of magma and undergoing four successive degassing events. **a**, relative mass of fluid released during each event as function of magma crystallinity. **b-f**, composition of the released fluids for each of the four events in term of salinity ($NaCl_{eq}$), copper, sulphur, zinc and lead contents plotted against magma crystallinity. Solid coloured lines are the density contours at the 67% and 95% confidence levels.

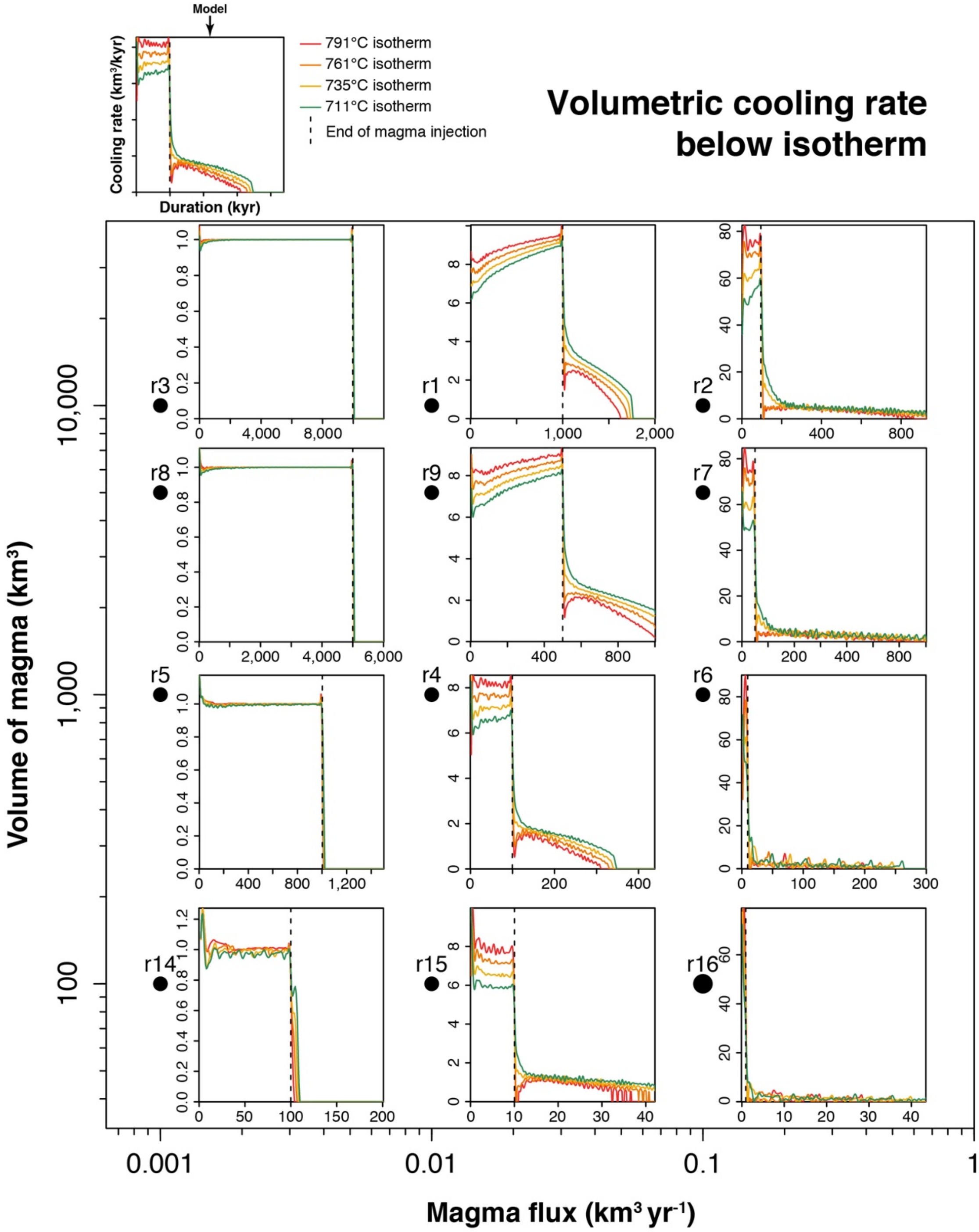

Supplementary Figure S2 | Volumetric cooling rate below the four considered isotherms for various magma fluxes and final injected volumes. Black points (rX) correspond to model runs for specified fluxes and volumes (see Supplementary Table S1).

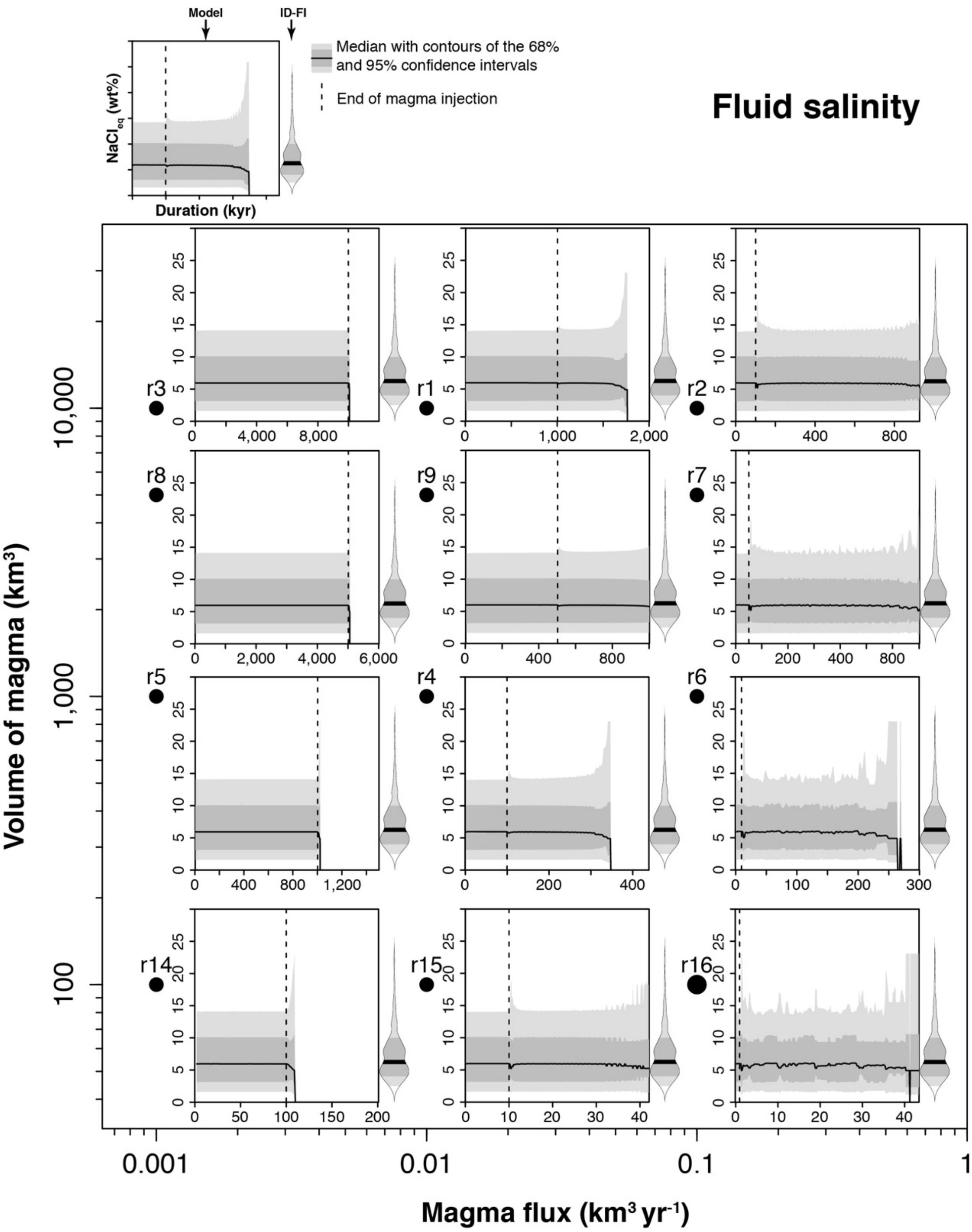

Supplementary Figure S3 | Computed fluid salinity for various magma fluxes and final injected volumes. The modelled fluid compositions are compared to fluid inclusion data from intermediate-density fluid inclusions (ID-FI) from porphyry deposits (from ref. 22) plotted as beans. Black points (rX) correspond to model runs for specified fluxes and volumes (see Supplementary Table S1).

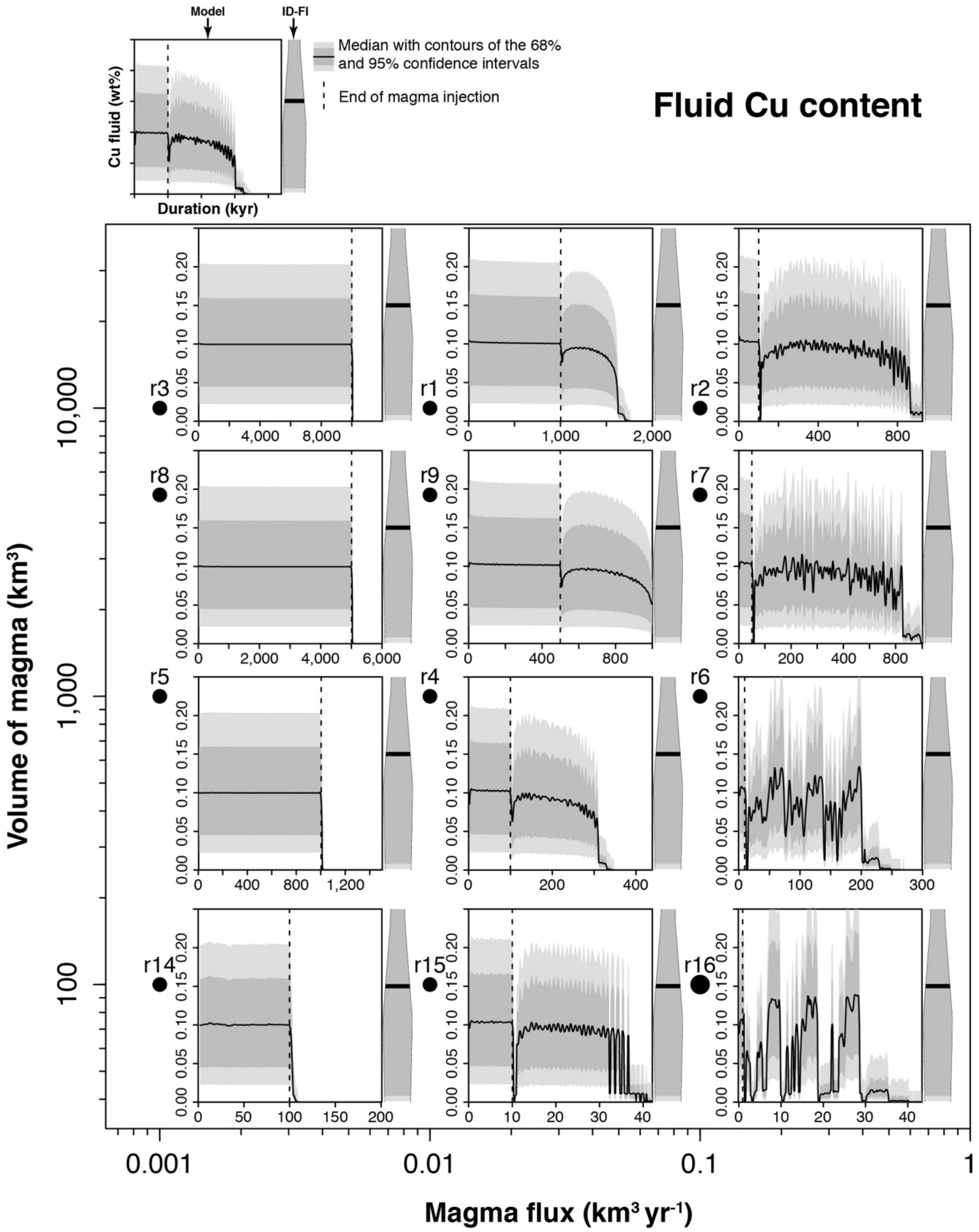

Supplementary Figure S4 | Computed fluid copper content for various magma fluxes and final injected volumes. The modelled fluid compositions are compared to fluid inclusion data from intermediate-density fluid inclusions (ID-FI) from porphyry deposits (from ref. 22) plotted as beans. Black points (rX) correspond to model runs for specified fluxes and volumes (see Supplementary Table S1).

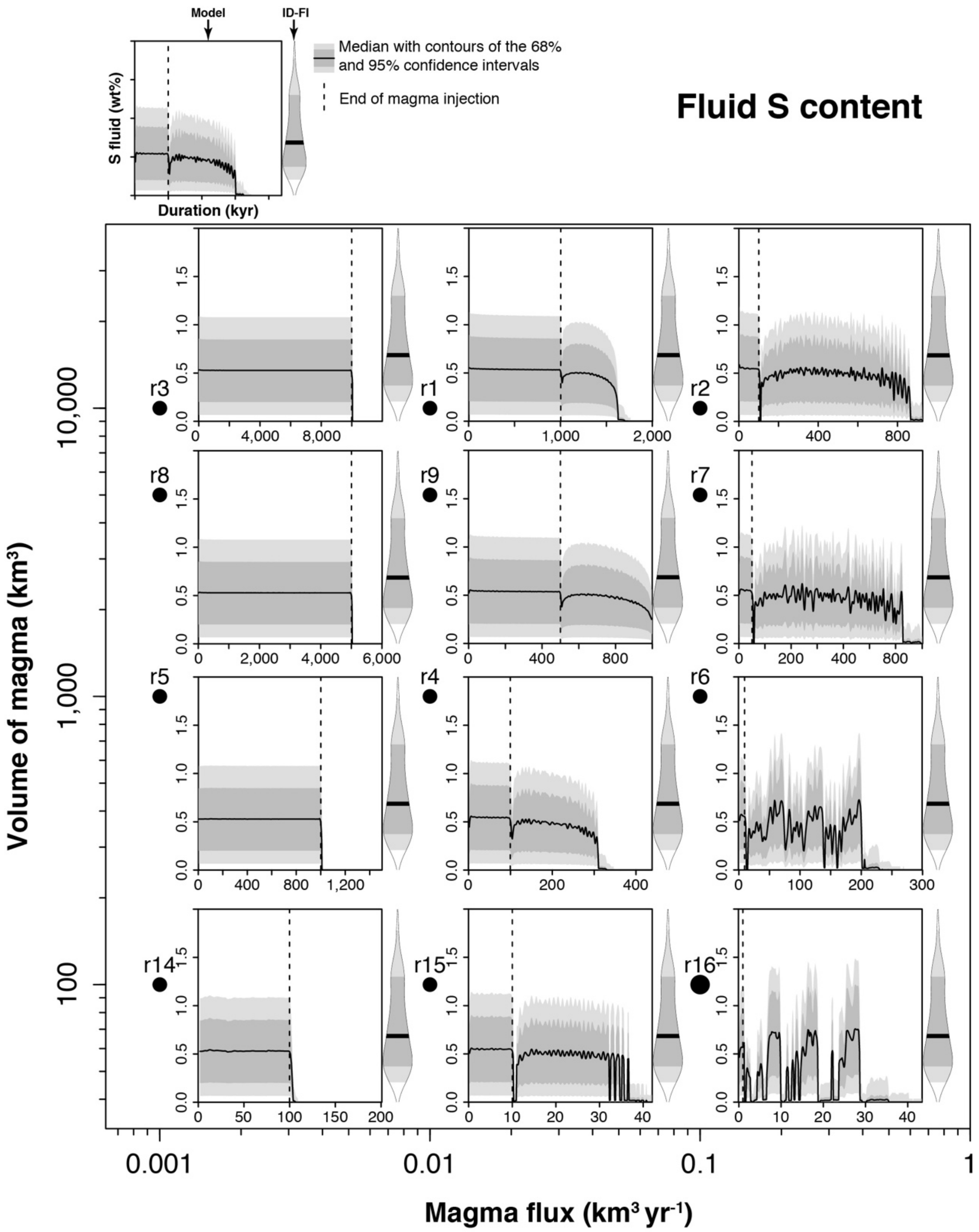

Supplementary Figure S5 | Computed fluid sulphur content for various magma fluxes and final injected volumes. The modelled fluid compositions are compared to fluid inclusion data from intermediate-density fluid inclusions (ID-FI) from porphyry deposits (from ref. 22) plotted as beans. Black points (rX) correspond to model runs for specified fluxes and volumes (see Supplementary Table S1).

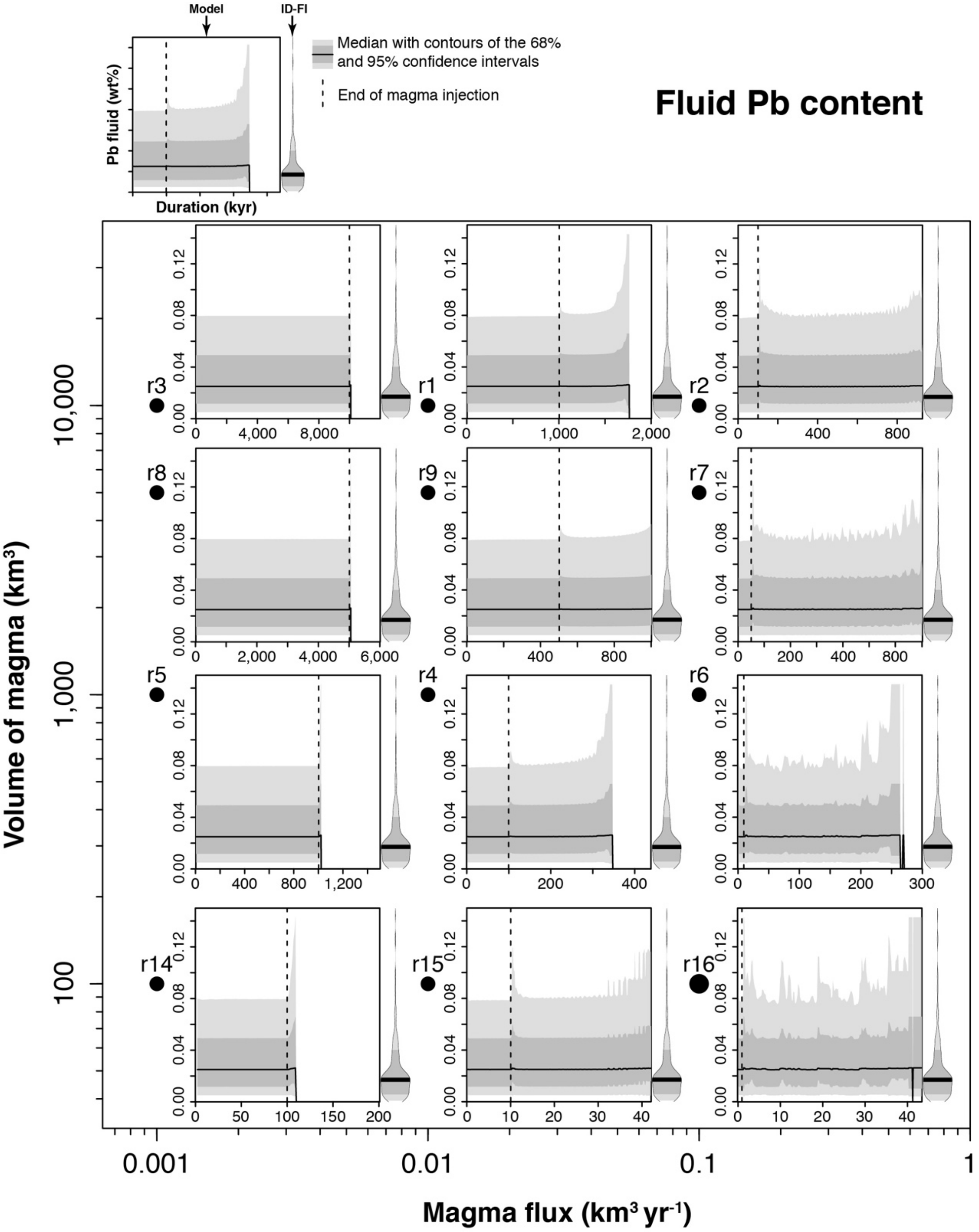

Supplementary Figure S6 | Computed fluid lead content for various magma fluxes and final injected volumes. The modelled fluid compositions are compared to fluid inclusion data from intermediate-density fluid inclusions (ID-FI) from porphyry deposits (from ref. 22) plotted as beans. Black points (rX) correspond to model runs for specified fluxes and volumes (see Supplementary Table S1).

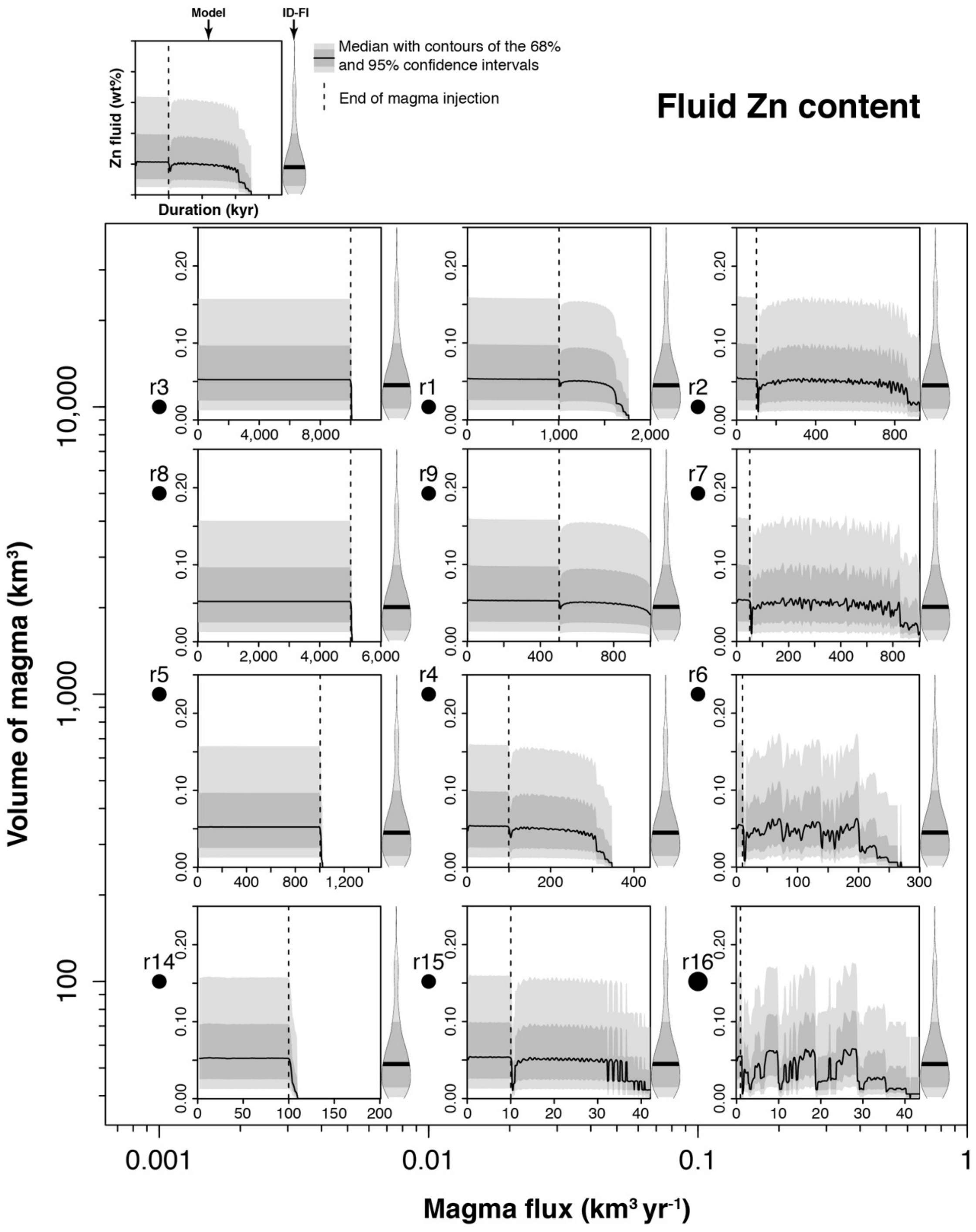

Supplementary Figure S7 | Computed fluid zinc content for various magma fluxes and final injected volumes. The modelled fluid compositions are compared to fluid inclusion data from intermediate-density fluid inclusions (ID-FI) from porphyry deposits (from ref. 22) plotted as beans. Black points (rX) correspond to model runs for specified fluxes and volumes (see Supplementary Table S1).

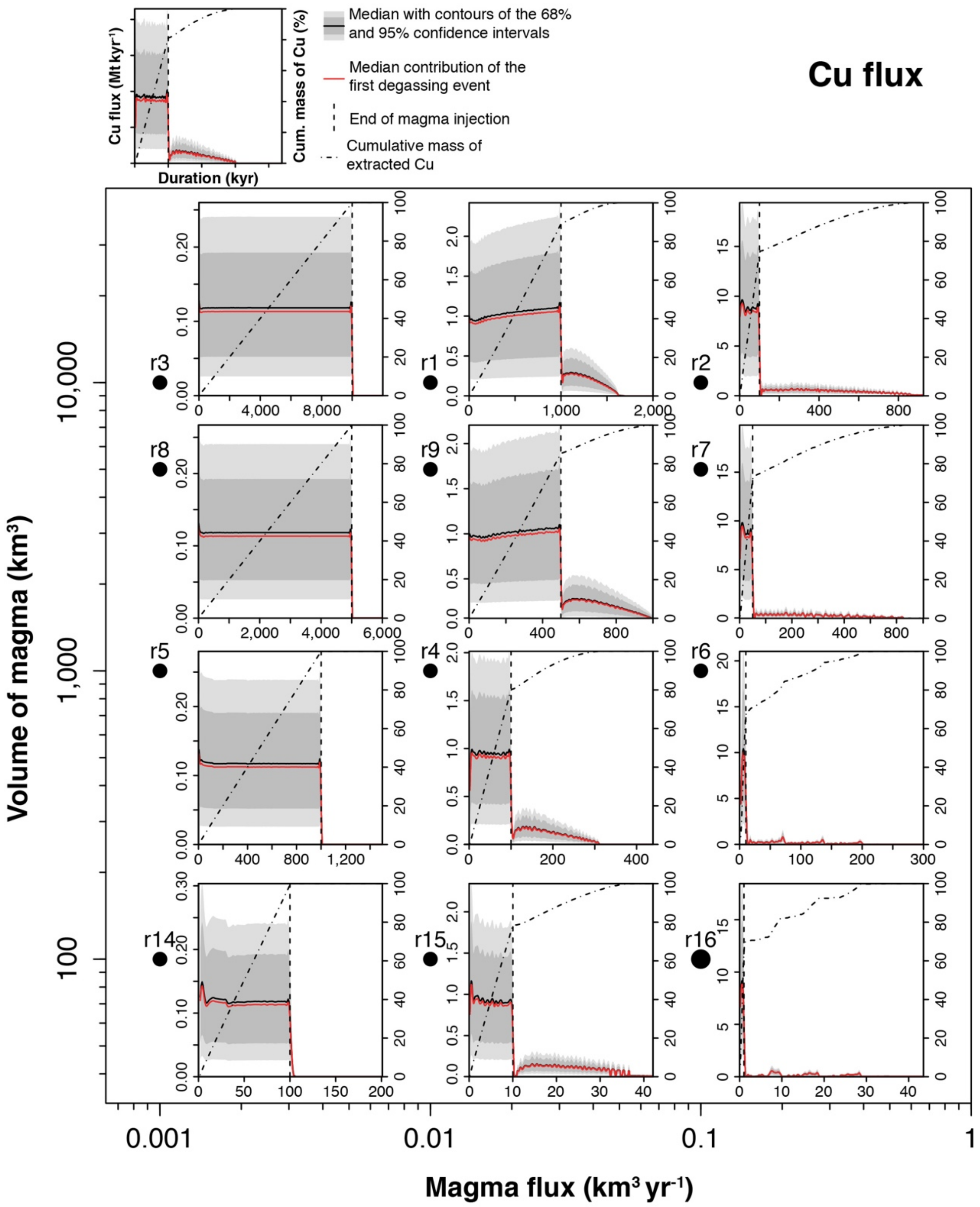

Supplementary Figure S8 | Computed copper flux for various magma fluxes and final injected volumes. Black points (rX) correspond to model runs for specified fluxes and volumes (see Supplementary Table S1).

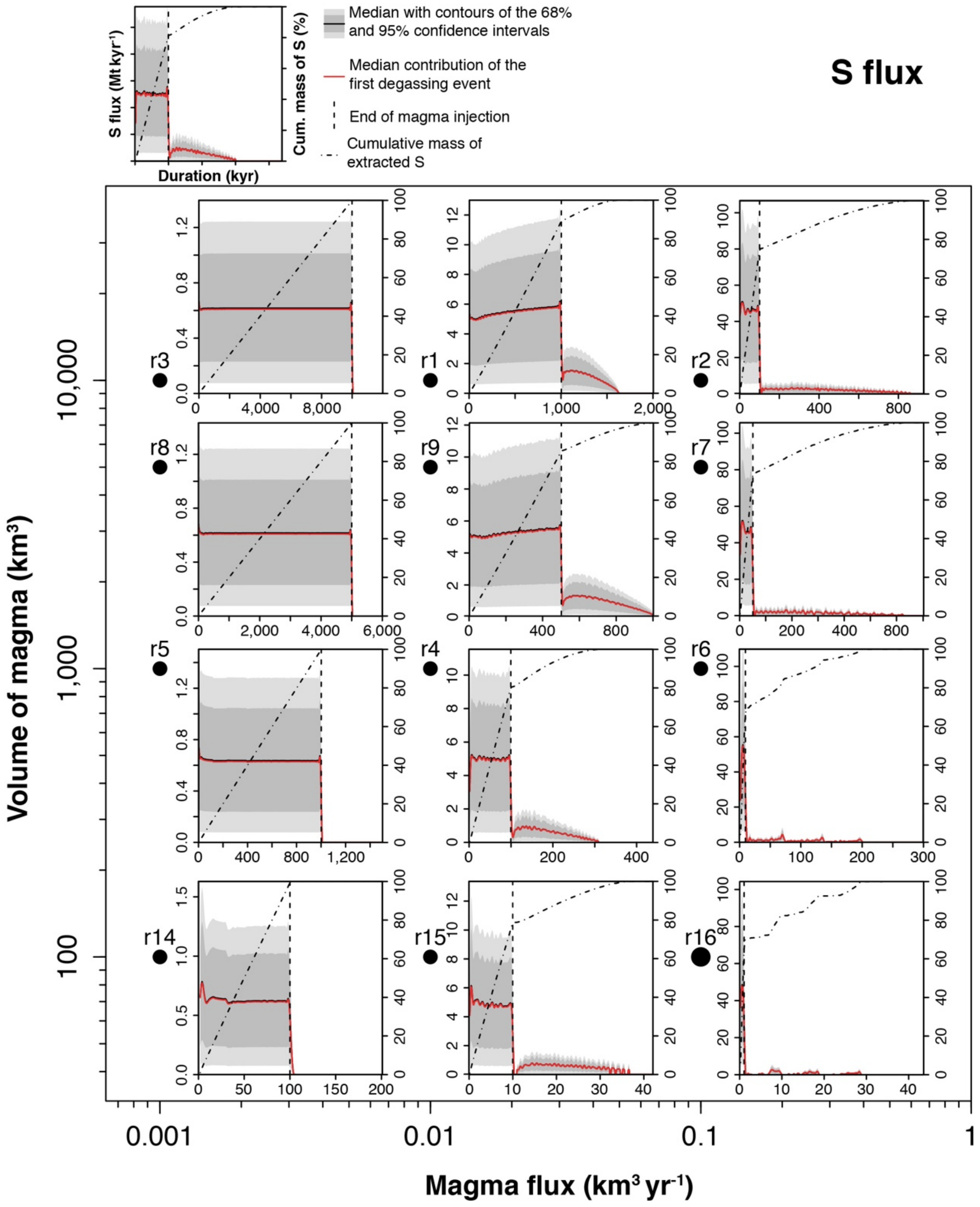

Supplementary Figure S9 | Computed sulphur flux for various magma fluxes and final injected volumes. Black points (rX) correspond to model runs for specified fluxes and volumes (see Supplementary Table S1).

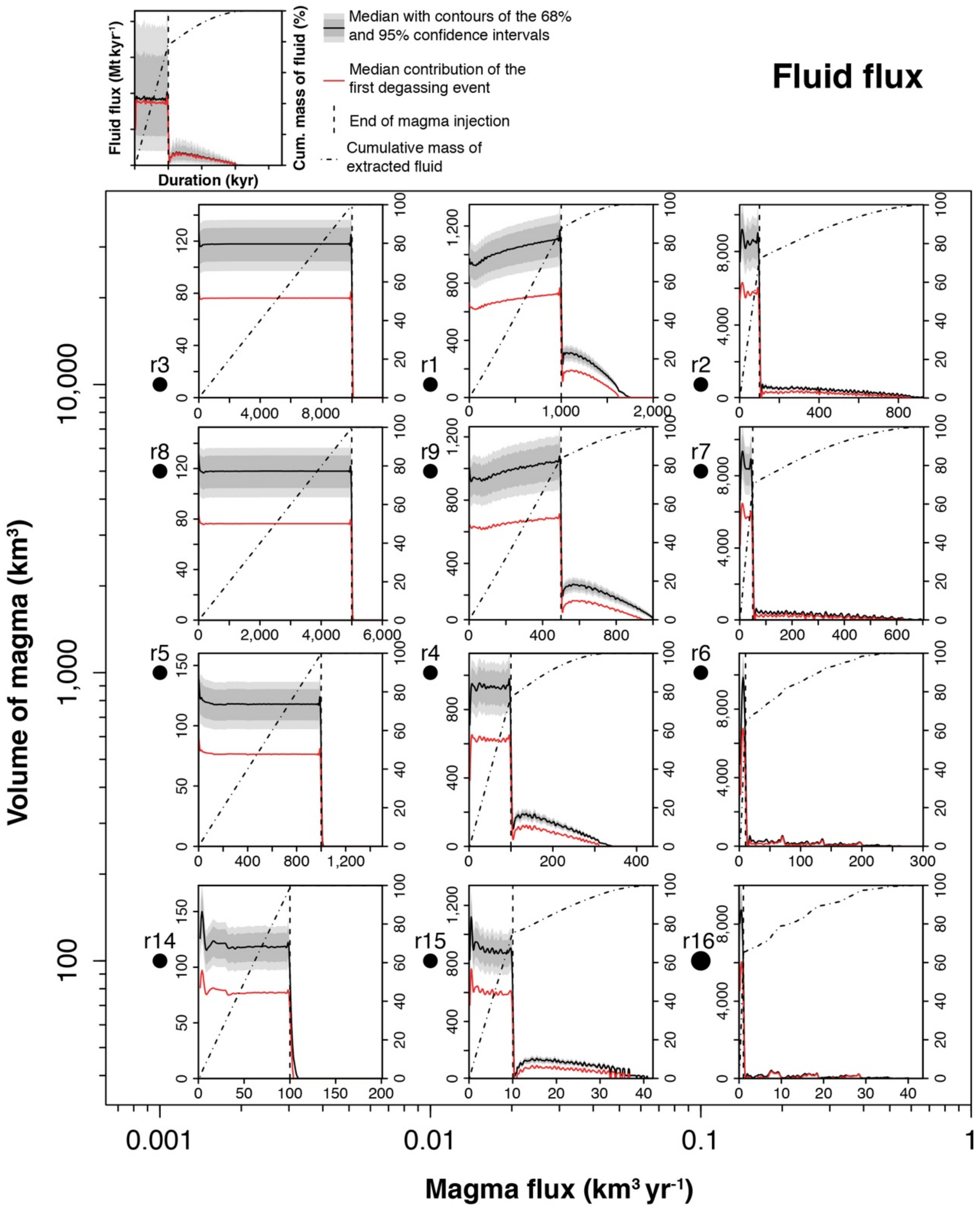

Supplementary Figure S10 | Computed fluid flux for various magma fluxes and final injected volumes. Black points (rX) correspond to model runs for specified fluxes and volumes (see Supplementary Table S1).

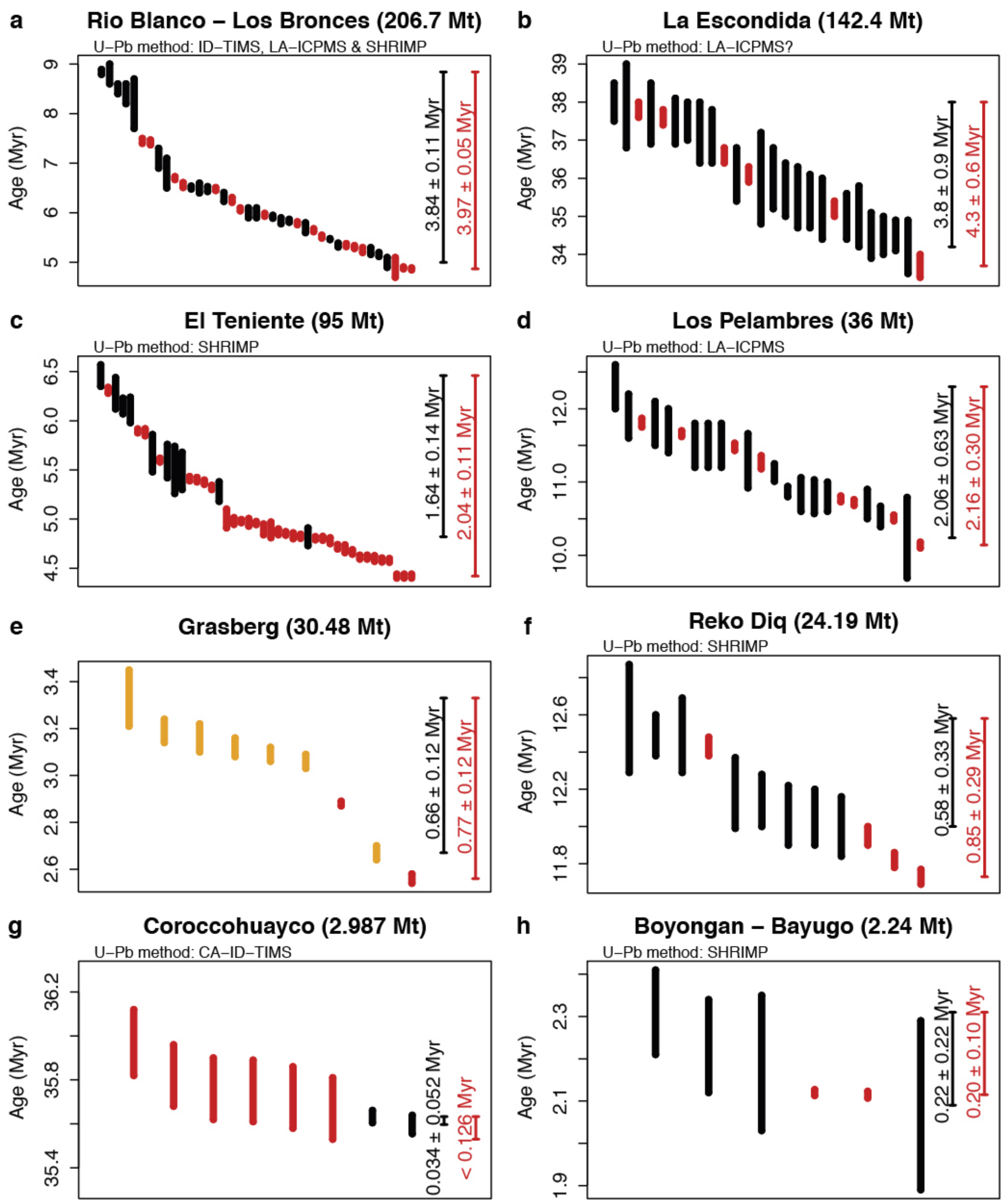

Supplementary Figure S11 | Compilation of radioisotopic geochronological data from well-constrained porphyry copper deposits. U-Pb zircon emplacement ages are in black, Re-Os molybdenite ages are in red and $^{40}Ar/^{39}Ar$ magmatic biotite ages (only for the Grasberg deposit) are in orange. The duration of the ore-related magmatic activity (black capped segments) and ore-forming fluid release (red capped segments) are shown on the right of each diagram. For each deposit, the copper endowment and the U-Pb method used for dating are provided.
